# Bisphosphonate compliance in Japan from the perspective of product, formulation, and patient characteristics: analysis of medical insurance claim data

**DOI:** 10.1186/s40780-025-00434-5

**Published:** 2025-04-08

**Authors:** Kenji Kabeya, Hiroki Satoh, Natsuyo Yanagi, Yasufumi Sawada

**Affiliations:** Laboratory of Drug Lifetime Management, Graduate School of Pharmaceutical Sciences, The University of Tokyo, 7-3-1 Hongo, Bunkyo-ku, Tokyo, 113-0033 Japan

**Keywords:** Bisphosphonate, Compliance, MPR (medication possession ratio), Claim data

## Abstract

**Background:**

Bisphosphonates are the mainstay drugs for osteoporosis, but in clinical practice, they are often ineffective due to low compliance. However, there have been few studies examining compliance on a product-by-product basis or in detail in Japan. This study aimed to clarify the bisphosphonate compliance from the viewpoints of product selection, formulation, and patient characteristics using medical insurance claim data in Japan, to generate useful knowledge for improving bisphosphonate compliance.

**Methods:**

Bisphosphonate records for osteoporosis treatment were extracted from Japanese medical insurance claim data (2021–2023), and the Medication Possession Ratio (MPR) of each patient was calculated from the records. The calculated MPR and compliance classification (Compliant/Non-compliant/Dropout) based on dispensing status were statistically analyzed from viewpoints of drug product, dose form/frequency, and patient sex/age to investigate the influence of each factor on compliance.

**Results:**

The mean MPR for all patients (*N* = 63,197) was 76.7%. Product choice influenced compliance, with significance in 230 pairs among the 71 major products. Tablet was the most compliant formulation, and compliance was better with longer dose intervals. Women showed significantly better compliance and older age was associated with better compliance.

**Conclusions:**

This study generated new data regarding product-specific MPRs, and clarified that product selection influences patient compliance. The study also supported previous findings that sex, age, and dose frequency influence compliance. It is expected that the findings of this study will be utilized for drug development, drug selection and patient guidance in clinical practice, to improve the treatment environment for osteoporosis.

**Supplementary Information:**

The online version contains supplementary material available at 10.1186/s40780-025-00434-5.

## Background

Bisphosphonates (BPs) are widely used worldwide as a primary treatment for osteoporosis, and previous studies have shown that medication compliance is crucial for achieving the desired therapeutic effect of BPs [1–3]. However, in clinical practice, compliance with BPs is generally poor, leading to diminished treatment outcomes and increased healthcare costs [4–6]. It is also known that even among drugs with the same efficacy and mechanism of action, compliance can vary depending on specific product characteristics and ease of use [7]. Traditionally, studies on compliance with BPs have focused on factors such as administration route, dosage frequency, or active ingredients [8–10]. However, although the information would be quite useful for physicians’ drug selection in clinical practice, there has been no detailed analysis of compliance differences among individual BP products at a finer granularity. Furthermore, studies examining these characteristics are limited in Japan, although its society is highly aged and a variety of BP products are widely used. Therefore, this study aims to elucidate the actual state of compliance with BPs in real-world clinical settings in Japan, from the viewpoints of product selection, formulation, and patient characteristics using medical insurance claim data.

## Methods

### Analyzed data

This study used medical insurance claims data collected and provided by JMDC Inc. (Tokyo, Japan). The data covers enrollees of society-managed employment-based health insurance in Japan, spanning ages 0 to 74, and represents approximately 8% of the entire population of Japan. The data, obtained from insurers, allows for cross-sectional tracking of each enrollee’s medical records, including diseases, prescriptions, and medical procedures. JMDC Inc. ensured that the data was sufficiently anonymized, and formal consent was not required for this retrospective study.

For the analysis, this study extracted records of BP prescriptions for osteoporosis patients from outpatient claims data spanning January 2021 to December 2023. The dataset was further refined by excluding data that met the following criteria (in italic):

- *Patients with a diagnosis of “Paget’s disease of bone.”* BPs used for Paget’s disease have different dosing regimens, making accurate Medication Possession Ratio (MPR) calculation difficult.
- *Records containing BPs NOT indicated for osteoporosis*. For example, although Zometa (zoledronate) is a BP, it is not indicated for osteoporosis. Records with etidronate dosages exceeding 400 mg/day were also excluded, as such dosages are not indicated for osteoporosis in Japan, suggesting usage for other diseases.
- *Records where the first BP prescription for each patient covered December 31*,* 2023*. If the first BP prescription covered December 31, 2023, the MPR would automatically be 100% due to the definition, negatively affecting accurate compliance measurement.
- *Records of the different product from the last dispensed BP for each patient*. If there was a change in the BP product used during the study period, records before the last change were excluded.

### Calculation of MPR

This study used the MPR as an indicator to assess medication compliance. MPR is the ratio of the sum of days supply to the total days in the period [11]. For instance, if the observation period is 100 days and a patient receives three 30-day prescriptions during the period, the MPR would be 90%.

MPR was calculated following the steps outlined in previous studies that computed MPR from claims data [11–13]. The observation period’s start date was defined as the date the patient first received the BP during the study period, and the end date was defined as the later of either the last prescription’s coverage end date or the final date the patient’s data was available in the claims. The sum of days supply was defined as the total coverage days of prescriptions received during the observation period. The coverage days for each prescription were calculated by multiplying the prescribed doses by the dosing frequency set according to the approved usage in Japan. For products with a “once a month” dosing regimen, 30 days of coverage was assumed, while a “once a year” regimen was considered to cover 365 days. For etidronate, which requires two weeks of continuous use followed by a 10–12 week drug-free period, a median break of 11 weeks was applied, equating to 6.5 days of coverage per dose.

### Analysis of MPR

To evaluate medication compliance for each patient, this study followed previous research and included not only the MPR calculation but also the classification into Compliant, Non-compliant, or Dropout categories [13]. “Dropout” was defined as cases where the interval between the last prescription’s coverage end date and the last available date in the claims data was 90 days or more. The other cases than “Dropout” were classified into two categories; “Compliant” was defined as cases where MPR was 80% or higher, and “Non-compliant” was defined as cases where MPR was less than 80%.

Both MPR and the distribution of Compliant/Non-compliant/Dropout categories were treated as dependent variables. Multivariable analyses were conducted with five independent variables: drug product, dose form, dose frequency, patient sex, and patient age (details below). MPR was analyzed using analysis of covariance (ANCOVA) and Tukey’s multiple comparison test, while the distribution of Compliant/Non-compliant/Dropout categories was analyzed using multinomial logistic regression. The effects of each independent variable were mutually adjusted, and the significance level was set at 5%.

- Drug product (For the analysis by drug product, products with fewer than 10 cases, and products whose sales termination had been announced by January 2024 were excluded. Since formulation and dosing frequency are characteristics associated with each product and cannot be separated in clinical practice, only patient age and sex were used as moderators in product-specific analyses.)
- Dose form: tablet, jerry, injection.
- Dose frequency: Daily, Weekly, Monthly, Yearly, Special (etidronate).
- Patient sex: Male, Female.
- Patient age: Under 40, 40s, 50s, 60s, 70+.

In analyzing claims data, we considered the potential impact of analysis period length on the results. One concern was that 2021 saw two declarations of a state of emergency due to the COVID-19 pandemic in Japan, which may have led to a decline in compliance due to reduced medical visits. Additionally, since BPs are commonly used for several years followed by a drug holiday period, extending the analysis period too long could result in a higher proportion of patients entering the drug holiday phase, potentially leading to an underestimation of medication compliance. To account for these potential differences, a sub-analysis was conducted using data limited to two years (from January 2022 to December 2023) in addition to the main analysis using the full three-year period (from January 2021 to December 2023).

### Tools for analysis

For data aggregation and analysis in this study, Alteryx Designer version 2024.1.1.17 and Microsoft Excel version 2406 were used, while IBM SPSS Statistics version 27.0.1.0 was used for statistical analysis.

## Results

### Data overview

After applying the selection criteria, data from 63,197 patients’ records were extracted and analyzed. The mean MPR for all patients was 76.7%. Among these patients, 41,303 (65.4%) were classified as Compliant, 5,004 (7.9%) as Non-compliant, and 16,890 (26.7%) as Dropout. The most commonly used active ingredient was alendronate, which was prescribed to 27,697 patients (Table 1).

### Compliance by patient characteristics

The results of ANCOVA and multinomial logistic regression analysis showed that, for both average MPR and Compliant/Non-Compliant/Dropout distribution, female patients had significantly better compliance compared to male patients (Table 1).

Regarding age groups, there was no significant effect on average MPR, but a significant effect was observed on Compliant/Non-Compliant/Dropout distribution. Compliance tended to improve with increasing age.

### Compliance by product/formulation

Dose form was shown to significantly influence MPR and compliance classification, with tablets demonstrating the highest MPR and Compliant rate, compared to jerry and injection (Table 1).

Dosing frequency also had a significant impact on MPR and compliance classification, with longer dosing intervals associated with better compliance. Etidronate, with its unique regimen, exhibited notably poor compliance. Tukey’s multiple comparison test revealed significant differences in all combinations except between Daily and Special dosing (Table 1).

**Table 1:** Medical compliance by patient and formulation characteristics

	*N*	MPR, mean (SD)	Compliant, %	Non-compliant, %	Dropout, %	Significant^b^ vs.
Overall	63,197	76.7% (33.4%)	65.4%	7.9%	26.7%	
Sex						
Male	12,960	73.0% (34.6%)	59.5%	8.8%	31.7%	Female
Female	50,237	77.7% (33.0%)	66.9%	7.7%	25.5%	Male
Age						N/A
Under 40	5,372	69.5% (36.6%)	54.4%	8.6%	37.0%	
40s	7,456	74.8% (34.2%)	62.1%	7.7%	30.2%	
50s	19,682	76.2% (33.8%)	64.8%	7.5%	27.7%	
60s	22,431	78.4% (32.0%)	68.1%	7.6%	24.3%	
70+	8,256	79.7% (32.3%)	69.1%	9.5%	21.3%	
Compound						N/A
ALN	27,697	75.3% (34.4%)	63.7%	7.8%	28.4%	
IBN	7,322	76.4% (32.4%)	64.0%	9.1%	26.9%	
MIN	13,562	77.0% (31.0%)	66.8%	8.6%	24.6%	
RIS	14,179	78.9% (34.2%)	67.6%	7.0%	25.4%	
ZOL	437	88.2% (18.5%)	75.3%	0.7%	24.0%	
ETD	30	56.3% (43.4%)	16.7%	13.3%	70.0%	
Dose form						
Tablet	53,082	76.9% (33.6%)	65.9%	7.7%	26.4%	Jerry, Injection
Jerry	5,363	75.5% (33.7%)	62.4%	8.0%	29.6%	Tablet
Injection	4,752	75.4% (30.9%)	63.0%	10.3%	26.7%	Tablet
Dose frequency						
Yearly	437	88.2% (18.5%)	75.3%	0.7%	24.0%	All the others
Monthly	27,704	77.7% (32.3%)	66.5%	8.3%	25.2%	All the others
Weekly	34,152	76.0% (34.2%)	64.6%	7.6%	27.8%	All the others
Daily	874	69.5% (37.4%)	57.7%	11.6%	30.8%	All but Special
Special^a^	30	56.3% (43.4%)	16.7%	13.3%	70.0%	All but Daily

MPR: Medication Possession Ratio, ALN: Alendronate, IBN: Ibandronate, MIN: Minodronate, RIS: Risedronate, ZOL: Zoledronate, ETD: Etidronate

^a^ Didronel (etidronate), ^b^ combinations where significance (*p* < 0.05 in ANCOVA for Sex, *p* < 0.05 in Tukey’s multiple comparison test for dose form/frequency) was observed on MPR; detailed statistical values are provided in Supplemental Table 1

A total of 71 products were selected for comparative analysis among individual drugs. The product most commonly used by patients was Bonalon oral jelly 35 mg. A large range was observed in MPR by product, with the highest MPR of 88.2% for Reclast IV 5 mg, compared to the lowest one of 56.3% for Didronel Tablet 200. The results of ANCOVA and multinomial logistic regression analysis indicated that drug choice significantly affected MPR and compliance classification. Tukey’s multiple comparison test found significant differences in MPR in 230 out of 2,485 product pairings, accounting for about 10% of the combinations (Table 2).

**Table 2:** Medical compliance by products (brand-name and major generic)

Product name	**Compound**	**Dose frequency**	**N**	MPR,	**Compliant**,	Non-compliant,	Dropout,	Superior to^a^, N	Inferior to^b^, N
				mean (SD)	%	%	%		
Reclast IV 5mg	ZOL	[Y]	437	88.2%(18.5%)	75.3%	0.7%	24.0%	43	0
Risedronate Tablet 75mg "Towa"	RIS	[M]	2887	82.1% (37.2%)	70.5%	6.5%	22.9%	23	0
Recalbon Tablet 1mg	MIN	[D]	23	80.7% (31.7%)	69.6%	8.7%	21.7%	0	0
Benet Tablet 75mg	RIS	[M]	1289	79.6% (33.1%)	67.2%	6.5%	26.3%	8	1
Benet Tablet 17.5mg	RIS	[W]	734	79.4% (32.4%)	69.6%	5.4%	24.9%	7	1
Actonel Tablet 75mg	RIS	[M]	1583	79.0% (33.4%)	66.3%	6.9%	26.8%	7	1
Minodronate Tablet 50mg "Sawai"	MIN	[M]	1826	78.8% (29.5%)	69.8%	8.7%	21.5%	7	1
Actonel Tablet 2.5mg	RIS	[D]	35	78.7% (34.3%)	68.6%	11.4%	20.0%	0	0
Fosamac Tablet 35mg	ALN	[W]	1449	78.6% (32.1%)	67.2%	7.1%	25.7%	7	1
Bonviva Tablet 100mg	IBN	[M]	3356	78.5% (33.1%)	66.2%	7.0%	26.8%	7	2
Minodronate Tablet 50mg "Towa"	MIN	[M]	2122	78.4% (30.6%)	68.8%	8.1%	23.2%	7	1
Actonel Tablet 17.5mg	RIS	[W]	1197	78.2% (32.8%)	66.4%	7.8%	25.8%	6	1
Minodronate Tablet 50mg "Nipro"	MIN	[M]	1967	78.1% (29.9%)	68.7%	8.6%	22.7%	7	1
Alendronate Tablet 35mg "Towa"	ALN	[W]	2718	77.5% (33.5%)	66.7%	7.4%	25.9%	7	2
Recalbon Tablet 50mg	MIN	[M]	1380	77.3% (31.0%)	65.7%	10.4%	23.8%	4	2
Risedronate Tablet 17.5mg "Sawai"	RIS	[W]	2288	77.2% (33.9%)	67.3%	6.8%	26.0%	4	2
Minodronate Tablet 50mg "Nichiiko"	MIN	[M]	1500	77.0% (30.2%)	67.1%	8.0%	24.9%	4	2
Alendronate Tablet 35mg "VTRS"	ALN	[W]	3299	76.8% (34%)	65.9%	7.9%	26.2%	4	3
Alendronate Tablet 35mg "Sawai"	ALN	[W]	3035	76.3% (33.8%)	64.8%	8.3%	26.9%	4	3
Alendronate Tablet 35mg "Nichiiko"	ALN	[W]	4484	76.1% (34.8%)	65.4%	6.9%	27.7%	4	3
Bonoteo Tablet 50mg	MIN	[M]	2339	76.0% (31.8%)	64.8%	8.9%	26.3%	4	3
Bonalon Jerry 35mg	ALN	[W]	5363	75.5% (33.7%)	62.4%	8.0%	29.6%	3	3
Bonalon Tablet 35mg	ALN	[W]	3024	75.4% (34.2%)	63.8%	7.6%	28.6%	2	3
Minodronate Tablet 50mg "YD"	MIN	[M]	1048	75.2% (32.3%)	65.8%	7.2%	27.0%	2	3
Benet Tablet 2.5mg	RIS	[D]	56	74.7% (38.7%)	71.4%	0.0%	28.6%	0	0
Bonviva Syringe IV 1mg	IBN	[M]	3479	73.7% (32.1%)	61.0%	10.3%	28.7%	1	12
Bonoteo Tablet 1mg	MIN	[D]	31	71.3% (34.7%)	54.8%	22.6%	22.6%	0	0
Bonalon Tablet 5mg	ALN	[D]	147	70.7% (37.2%)	59.2%	9.5%	31.3%	0	1
Bonalon IV 900µg	ALN	[M]	314	69.0% (31.2%)	57.6%	14.6%	27.7%	0	16
Didronel Tablet 200	ETD	[S]	30	56.3% (43.4%)	16.7%	13.3%	70.0%	0	5

This table contains generic drugs with 1,000+ cases and brand-name drugs only among 71 products found in the analyzed claims data

MPR: Medication Possession Ratio, IV: Intravenous (Injection), ALN: Alendronate, IBN: Ibandronate, MIN: Minodronate, RIS: Risedronate, ZOL: Zoledronate, ETD: Etidronate, [D]: Daily, [W]: Weekly, [M]: Monthly, [Y]: Yearly, [S]: Special (two weeks of continuous daily use followed by a 10-12 week break)

^a^ the number of products which had significantly lower MPR than this product (among all the 71 products), ^b^ the number of products which had significantly higher MPR than this product (among all the 71 products)

### Influence of analysis period

Even when the analysis was limited to a two-year period, similar trends were observed for MPR and Compliant/Non-Compliant/Dropout distribution, with only slight differences in statistical significance (Supplemental Tables 2 and 3).

## Discussion

Overall, the MPR and Compliant rate observed in this study were higher (7–12% points in MPR, 10–29% points in compliant rate) than those reported in previous studies [5, 6, 8, 11, 12]. One possible explanation is that the observation period in this study was relatively short due to the limitations of the claims data, potentially not capturing the gradual decline in compliance that may occur with long-term use. Additionally, the proportion of Non-compliant patients was low, indicating that patients with poor compliance were more likely to completely drop out of treatment rather than continue with low adherence.

### Compliance by product/formulation

When comparing across products, there was significant variability in MPR and Compliant/Non-Compliant/Dropout distribution, suggesting that the most frequently used drugs in clinical practice are not necessarily those with the best compliance. Considering that 71 drugs were compared using Tukey’s multiple comparison test, which lowers the statistical power, it is speculated that drug selection has an even greater impact on compliance than indicated by this analysis. While factors such as efficacy are also important and cannot be generalized, the widespread use of drugs with lower compliance could lead to patient disadvantages and societal costs. Therefore, careful attention to drug selection is essential to enhance patient compliance and maximize therapeutic effects, in addition to considerations of formulation and dosing frequency.

For dose form, tablets showed better compliance than jerry or injection. This result contradicts previous studies suggesting that compliance with injectable formulations is better than with oral formulations [9, 14]. In this study, the dose form factor (tablet/jerry/injection) was isolated and examined, whereas most previous studies focusing on dose forms took an approach based on “replacing weekly oral formulations with monthly/yearly injectable formulations.” Thus, it is plausible that the conclusions of previous studies were more influenced by dosing intervals rather than the dose form itself, which may explain the discrepancy with the results of this study. It is also noteworthy that jerry formulations are often prescribed to patients with difficulty in swallowing, which can lower compliance. This patient factor cannot be adjusted for sex or age and may, therefore, significantly impact compliance with jerry formulations. Additionally, there is only one jerry product, Bonalon oral jelly 35 mg, meaning that the results may be more reflective of the product itself rather than the dose form. Therefore, a more detailed examination of the compliance characteristics of jerry formulations would require more patient data and an environment where at least several jerry products are available. It is also important to consider the impact of leftover medication for oral formulations. For example, even if patients receive prescriptions at appropriate intervals, they may not take them correctly at home, leading to the accumulation of leftover medication. This situation does not occur with injectable formulations, suggesting that actual compliance may be worse for oral formulations.

Dose frequency was significantly associated with MPR and Compliant/Non-Compliant/Dropout distribution, with longer intervals correlating with better compliance. This result aligns with previous studies, and this study was able to comprehensively confirm the relationship between dosing frequency and compliance, ranging from daily to yearly dosing [8, 9, 12, 15]. Notably, etidronate had poorer compliance compared to all other BP regimens, including daily dosing. Though the limited number of cases (30 cases out of 63,197 cases) might have influenced the result, this is likely due to the complexity of its regimen, which involves two weeks of continuous daily use followed by a 10–12 week break.

### Compliance by patient characteristics

Regarding patient characteristics, female patients exhibited better compliance than male patients. This trend has also been observed in previous studies not only in the context of osteoporosis or bisphosphonates but also in other therapeutic areas [11, 13, 16].

Furthermore, compliance tended to improve with age. This finding is also consistent with previous studies, possibly due to higher disease awareness or more available time for elderly patients [11, 13]. Given the nature of osteoporosis, where repeated fragility fractures in younger patients increase the risk of future fractures and significantly impair functional prognosis, focused follow-up on compliance may be particularly necessary for younger and male patients. This includes developing and distributing disease awareness and patient education materials specifically targeted at these groups, and enhancing medication counseling by community pharmacists for them.

### Influence of analysis period

Analysis limited to a two-year period did not change the trends observed with the five independent variables, indicating that the analysis period had minimal impact on the conclusions.

### Limitation

This study has several limitations. First, the claims data do not include patients aged 75 and older; therefore, if there are further substantial differences in compliance trends by age, the study may not have fully captured the impact on elderly patients. Additionally, due to the nature of the data, it was not possible to capture legitimate discontinuations of medication due to the physician’s decision or the presence of leftover medication, leaving some inaccuracy in the estimation of compliance. Furthermore, there may be limitations related to the tracking/analysis period within the claims data; in particular, for drugs with long dosing intervals, such as zoledronate, it may not have been possible to observe a sufficient number of cycles, potentially overestimating the MPR.

## Conclusions

This study used medical insurance claims data in Japan to investigate the actual state of bisphosphonate compliance in clinical practice, focusing on product selection, formulation, and patient characteristics.

The findings revealed that compliance with BPs in clinical practice in Japan is influenced by factors such as drug choice, formulation characteristics, and patient characteristics. The comprehensive product-specific MPR data presented in this study offer novel insights. Unlike previous findings, it was suggested that tablets might have better compliance than jerry or injection. Consistent with prior studies, longer dosing intervals were associated with better compliance, and female and elderly patients showed higher compliance.

The results of this study provide several implications for osteoporosis treatment using BPs. For example, the relationship between formulation characteristics and compliance may inform future BP development and improvement. The relationship between patient characteristics and compliance offers practical guidance to physicians and pharmacists in clinical settings on which patients may require more attention to compliance. Moreover, by comparing compliance at the product level, this study provides foundational data that could inform drug selection in clinical practice and identify the most cost-effective BP from a health economic perspective.

## Electronic supplementary material

Below is the link to the electronic supplementary material.

Supplementary Material 1

## Data Availability

The data that support the findings of this study are available from JMDC Inc. but restrictions apply to the availability of these data, which were used under license for the current study, and so are not publicly available. Data are however available from the authors upon reasonable request and with permission of JMDC Inc.

## Abbreviations

BP: Bisphosphonate
MPR: Medication possession ratio
ANCOVA: Analysis of covariance
