# Supplementary material for: Bisphosphonate compliance in Japan from the perspective of product, formulation, and patient characteristics: analysis of medical insurance claim data
Source: J Pharm Health Care Sci. 2025 Apr 8;11:30. doi: 10.1186/s40780-025-00434-5 (PMC11980115; doi:10.1186/s40780-025-00434-5)
Supplement: Supplementary file 1 — Supplementary Material 1 [file 40780_2025_434_MOESM1_ESM.docx]

**Supplemental Table 1. Statistical Values from Tukey’s multiple comparison test on MPR (3-year analysis)**

| **Characteristic** | **vs.** | **Difference [95% CI]** | **Standard Error** | **P value** |
| --- | --- | --- | --- | --- |
| Daily | Weekly | -0.0644 [-0.0955 - -0.0333] | 0.0114 | <0.001 |
|  | Monthly | -0.0819 [-0.1130 - -0.0506] | 0.0114 | <0.001 |
|  | Yearly | -0.1872 [-0.2403 - -0.1340] | 0.0195 | <0.001 |
|  | Special | 0.1318 [-0.0362 - 0.3000] | 0.0616 | 0.204 |
| Weekly | Monthly | -0.0174 [-0.0247 - -0.0101] | 0.0026 | <0.001 |
|  | Yearly | -0.1228 [-0.1664 - -0.0791] | 0.0160 | <0.001 |
|  | Special | 0.1962 [0.0308 - 0.3616] | 0.0606 | 0.011 |
| Monthly | Yearly | -0.1054 [-0.1491 - -0.0616] | 0.0160 | <0.001 |
|  | Special | 0.2137 [0.0483 - 0.3791] | 0.0606 | 0.004 |
| Yearly | Special | 0.3190 [0.1481 - 0.4899] | 0.0626 | <0.001 |
| Tablet | Jerry | 0.0143 [0.0031 - 0.0254] | 0.0048 | 0.007 |
|  | Injection | 0.0151 [0.0033 - 0.0269] | 0.0050 | 0.008 |
| Jerry | Injection | 0.0008 [-0.0147 - 0.0163] | 0.0066 | 0.992 |

CI: Confidence Interval, MPR: Medication Possession Ratio, Special: two weeks of continuous daily use followed by a 10-12 week break

**Supplemental Table 2. Medical compliance by patient and formulation characteristics (2-year analysis)**

|  | **N** | **MPR,  mean (SD)** | **Compliant, %** | **Non-compliant,  %** | **Dropout,  %** | **Significant^b^ vs.** |
| --- | --- | --- | --- | --- | --- | --- |
| Overall | 52,351 | 80.7% (30.5%) | 70.6% | 8.2% | 21.2% |  |
| Sex |  |  |  |  |  |  |
| Male | 10,726 | 77.6% (32.3%) | 65.7% | 9.0% | 25.3% | Female |
| Female | 41,625 | 81.5% (29.9%) | 71.8% | 8.0% | 20.2% | Male |
| Age |  |  |  |  |  | N/A |
| Under 40 | 4,472 | 74.5% (34.3%) | 60.6% | 9.3% | 30.1% |  |
| 40s | 6,454 | 79.3% (31.6%) | 67.8% | 8.1% | 24.1% |  |
| 50s | 17,253 | 80.4% (31.0%) | 70.5% | 7.6% | 21.9% |  |
| 60s | 18,336 | 82.2% (29.2%) | 73.0% | 8.0% | 19.0% |  |
| 70+ | 5,836 | 83.3% (27.4%) | 73.9% | 9.9% | 16.3% |  |
| Dose form |  |  |  |  |  |  |
| Tablet | 44,060 | 81.0% (30.6%) | 71.1% | 7.9% | 21.0% | Injection |
| Jerry | 4,478 | 79.9% (30.9%) | 68.2% | 8.4% | 23.4% | None |
| Injection | 3,813 | 78.8% (28.8%) | 67.7% | 11.1% | 21.2% | Tablet |
| Dose frequency |  |  |  |  |  |  |
| Yearly | 319 | 92.2% (13.2%) | 84.3% | 0.0% | 15.7% | All the others |
| Monthly | 22,770 | 81.4% (29.2%) | 71.4% | 8.7% | 19.9% | All the others |
| Weekly | 28,530 | 80.3% (31.5%) | 70.0% | 7.8% | 22.2% | All the others |
| Daily | 711 | 74.4% (34.7%) | 62.3% | 12.8% | 24.9% | All the others |
| Special^a^ | 21 | 52.5% (31.6%) | 23.8% | 9.5% | 66.7% | All the others |

MPR: Medication Possession Ratio

^a^ Didronel (etidronate), ^b^ combinations where significance (p<0.05 in ANCOVA for Sex, p<0.05 in Tukey’s multiple comparison test for dose form/frequency) was observed on MPR

**Supplemental Table 3. Statistical Values from Tukey’s multiple comparison test on MPR (2-year analysis)**

| **Characteristic** | **vs.** | **Difference [95% CI]** | **Standard Error** | **P value** |
| --- | --- | --- | --- | --- |
| Daily | Weekly | -0.0583 [-0.0897 - -0.0269] | 0.0115 | <0.001 |
|  | Monthly | -0.0698 [-0.1012 - -0.0383] | 0.0115 | <0.001 |
|  | Yearly | -0.1778 [-0.2335 - -0.1221] | 0.0204 | <0.001 |
|  | Special | 0.2197 [0.0367 - 0.4027] | 0.0671 | 0.009 |
| Weekly | Monthly | -0.0114 [-0.0188 - -0.041] | 0.0027 | <0.001 |
|  | Yearly | -0.1195 [-0.1660 - -0.0729] | 0.0171 | <0.001 |
|  | Special | 0.2780 [0.0976 - 0.4585] | 0.0662 | <0.001 |
| Monthly | Yearly | -0.1080 [-0.1546 - -0.0614] | 0.0171 | <0.001 |
|  | Special | 0.2895 [0.1090 - 0.4699] | 0.0662 | <0.001 |
| Yearly | Special | 0.3975 [0.2112- 0.5837] | 0.0683 | <0.001 |
| Tablet | Jerry | 0.0110 [-0.0001 - 0.0222] | 0.0048 | 0.053 |
|  | Injection | 0.0222 [0.0103 - 0.0342] | 0.0051 | <0.001 |
| Jerry | Injection | 0.0112 [-0.0044 - 0.0269] | 0.0067 | 0.214 |

CI: Confidence Interval, MPR: Medication Possession Ratio, Special: two weeks of continuous daily use followed by a 10-12 week break
